# The Changing Face of Mastectomy (from Mutilation to Aid to Breast Reconstruction)

**DOI:** 10.1155/2011/980158

**Published:** 2011-06-05

**Authors:** Stefano Zurrida, Fabio Bassi, Paolo Arnone, Stefano Martella, Andres Del Castillo, Rafael Ribeiro Martini, M. Eugenia Semenkiw, Pietro Caldarella

**Affiliations:** ^1^Division of Senology, European Institute of Oncology, 20141 Milan, Italy; ^2^School of Medicine, University of Milan, 20141 Milan, Italy; ^3^Division of Plastic Surgery, European Institute of Oncology, 20141 Milan, Italy

## Abstract

Breast cancer is the most common cancer in women. Primary treatment is surgery, with mastectomy as the main treatment for most of the twentieth century. However, over that time, the extent of the procedure varied, and less extensive mastectomies are employed today compared to those used in the past, as excessively mutilating procedures did not improve survival. Today, many women receive breast-conserving surgery, usually with radiotherapy to the residual breast, instead of mastectomy, as it has been shown to be as effective as mastectomy in early disease. The relatively new skin-sparing mastectomy, often with immediate breast reconstruction, improves aesthetic outcomes and is oncologically safe. Nipple-sparing mastectomy is newer and used increasingly, with better acceptance by patients, and again appears to be oncologically safe. Breast reconstruction is an important adjunct to mastectomy, as it has a positive psychological impact on the patient, contributing to improved quality of life.

## 1. Introduction

Escharotic materials and cautery were the main treatments for breast cancer for hundreds years. Various simple mastectomy procedures were introduced during the nineteenth century, but none was able to cure the disease or achieve adequate disease control [1].

## 2. Radical Mastectomy

The modern era of surgical treatment for primary breast cancer began in the 1890s with the introduction of radical mastectomy by Halsted and Meyer. William Stewart Halsted reported his work briefly in 1890 and published his more extensive experience of patients treated at Johns Hopkins Hospital, Baltimore, in November 1894 [2]. Willy Meyer published his experience of New York patients in December 1894 [3]. Some of Halsted and Meyer's patients were cured of their disease, so for the first time, physicians had an effective treatment for breast cancer, and radical mastectomy quickly became the standard of care for the disease. 

### 2.1. The Halsted-Meyer Theory

Radical mastectomy was based on the idea that at first, breast cancer spread only locally or “centrifugally” by first invading contiguous tissue and then spreading though lymph ducts to close-by lymph nodes, where the cells were “trapped” for some time. Haematic spread of tumour cells—giving rise to distant metastases—was considered to occur at a later stage.

### 2.2. Features of Radical Mastectomy

Radical mastectomy involves the removal of all breast tissue, overlying skin, and both pectoralis muscles, together with complete en bloc removal of the axillary lymph nodes. Skin was removed because the disease often involved the skin; in fact, the skin was often ulcerated on presentation [2, 4]. The pectoralis muscles were removed not simply because the chest wall was often involved, but because it was considered essential to remove the transpectoral lymphatic pathways that run directly through the pectoralis major to Rotter's nodes between the pectoralis major and pectoralis minor. At that time, it was also considered anatomically impossible to do a complete axillary dissection without removing the pectoralis muscles [2, 3]. 

Halsted achieved a three-year local recurrence rate of 3% and locoregional recurrence rate of 20% with no perioperative mortality. Five-year survival was 40%—twice that of untreated patients [2]. However, morbidity after the operation was great, because the large wounds were left to heal by granulation, lymphedema was near universal, and arm movement was severely restricted (due to pectoralis muscle removal and damage to axilla nerves). For these reasons, chronic pain was also an important sequela. Over a century ago, surgeons were faced with large breast cancers that seemed to require drastic treatment to have some chance of cure: patients' quality of life was not a consideration [4–10].

Nevertheless, thanks to Halsted and Meyer, at last, it became possible to cure breast cancer in some cases, and systematic knowledge of the disease began to accumulate, standardized treatments started being applied, and controlled long-term studies would eventually be conducted. It also became apparent that some women with advanced disease did not benefit from surgical treatment, and as the twentieth century advanced, the concepts of operability and inoperability were developed, largely by Haagensen and Stout [11]. 

From 1930 onward, orthovoltage radiotherapy was added to mastectomy in many treatment centers although the radiation dose and size and location of treatment fields varied considerably [12–14].

### 2.3. Extended Radical Mastectomies

According to the Halsted-Meyer theory the most important pathway for breast cancer dissemination was through the lymphatic ducts. The implication was that chances of cure were increased by performing wider (and ever more mutilating) surgical resections that removed more lymph nodes [15]. From 1920 onwards, Samson Handley employed an “extended” radical mastectomy that included removal of the lymph nodes of the internal mammary chain. He also implanted radium needles into the anterior intercostal spaces in order to treat internal mammary nodes. Richard Handley, Samson Handley's son, studied internal mammary chain nodal involvement in breast cancer, demonstrating that 33% of 150 breast cancer patients had internal mammary chain involvement at the time of surgery [16]. Andbeassen et al. [17] in Denmark and Margottini [18] in Italy performed extrapleural dissection of the internal mammary nodes [19], while Wangensteen [20] in the USA performed supraclavicular and internal mammary node dissection through a sternal approach. Urban [21], at the Memorial Sloan-Kettering Hospital of New York, performed a massive 4-5 hour operation that combined radical mastectomy with resection of the internal mammary chain en bloc with costal cartilages and intercostal muscles; defects were made good with a fascia lata graft.

## 3. Reduced Mastectomies

### 3.1. The Patey-Dyson Mastectomy

The centrifugal spread idea held sway throughout the first half of the 20th century, and progress in breast cancer treatment was considered, at least by some [22], to depend on even more radical ablations. But perhaps the first indication that extended radical mastectomies were not necessary came from a study by Gray [23] published in 1940, which showed that the dermis was rich in lymphatic vessels and likely to be a plane of ready cancer spread, while the fascia underlying the breast was practically devoid of lymphatic vessels, which for this reason was unlikely to be a major plane of cancer spread. These findings encouraged some surgeons to remove more skin; however, Patey and Dyson [24] experimented with a reduced mastectomy that preserved the pectoralis major: in their 1948 paper, they reviewed mastectomies performed between 1930 and 1943, comparing their operation (which spared the pectoralis major) with the standard radical mastectomy. They found no difference in survival or local recurrence rates between the two groups. Patey wrote “Until an efficient general agent for the treatment of carcinoma of the breast is developed, a high proportion of cases are doomed to die of the disease whatever combination of local treatment by surgery and irradiation is used, because in such a high proportion of cases the disease has passed outside the field of local attack when the patient first comes for treatment.”

### 3.2. The Madden-Auchincloss Mastectomy

In 1972, Madden and colleagues [25] presented results of a consecutive series of patients treated by their “modified” radical mastectomy, in which both the pectoralis major and the pectoralis minor were preserved. Outcomes were similar to those using the radical mastectomy. Patey argued that complete axillary dissection was not possible if the pectoralis minor was preserved. It was also argued that the pectoralis minor's nerve and blood supply were not conserved, leaving the muscle useless [24]. 

However, Madden's lymphangiographic data showed it was possible to clear the axilla. It was also possible to preserve the neurovascular supply to the pectoralis minor muscle [25]. Auchincloss [26] also presented data in favour of the modified radical mastectomy that conserved the pectoralis minor. Auchincloss also questioned the need to perform complete axillary dissection, suggesting that the apical nodes (Berg level III—[27]) should only be removed when evidently invaded. 

Crile [28] went further and suggested that axillary dissection should not be performed immediately if the axilla was not evidently involved, at least in patients with stage I breast cancer, but only subsequently if axillary involvement developed. Survival rates following delayed axillary dissection were equal to or better than following prophylactic lymph node dissection [28]. 

Another objection to preservation of the pectoralis minor was that the interpectoral lymphatic (Rotter) nodes could be a source of disease recurrence. However, recurrence at this site is unusual, and even if it occurs, muscle invasion is uncommon.

### 3.3. Simple Mastectomy

The simple mastectomy was first developed by Kennedy and Miller [29], based on indications that radical mastectomy was not always necessary in women with breast cancer. Simple mastectomy is a fairly rapid operation in which the pectoral fascia is removed in bloc with the breast, but neither the pectoralis muscles nor the axillary lymph nodes are removed. Kaae and Johansen [30] compared simple mastectomy plus postoperative radiotherapy with extended radical mastectomy plus radiotherapy and found that overall survival rates were similar in both.

## 4. Breast Conservation as an Alternative to Mastectomy

The controversy between extended and reduced mastectomies crystallized in the 1970s when Bernard Fisher marshalled evidence that breast cancer was a systemic disease from the outset and that distant metastases were present well before diagnosis in most cases. The implication was that extended mastectomies were useless. However, Fisher also cited evidence that debulking the tumor mass might stimulate the body to destroy remnant tumor cells by immunologic and other mechanisms, perhaps in combination with systemic cytotoxic agents [31]. These ideas stimulated surgeons to experiment with breast-conserving treatments for breast cancer, variably combined with elective axillary dissection, radiotherapy to the residual breast, and chemotherapy. The first major clinical trials investigating breast conservation began in the 1970s, in coincidence with more widespread use of mammography to identify small lesions and permit diagnosis of breast cancer at an earlier stage. The first to trial to publish five-year results was that of Veronesi's group in Milan [32]. This trial compared quadrantectomy plus radiotherapy plus axillary dissection with radical mastectomy and found no difference between the two treatments. Subsequently, Fisher et al. [33] published five-year results of their trial comparing lumpectomy with “total mastectomy” that removed the entire breast, pectoral fascia, and axillary contents en bloc. Twenty-year follow-up of both these landmark studies [34, 35] confirmed that conservative breast surgery is equivalent to mastectomy as a treatment for breast cancer. This was an important advance, as the mutilation that went with mastectomy was considerably reduced by the conservative approach.

## 5. New Mastectomies

Today the standard of care for patients with stage I/II breast cancer is lumpectomy or a more extended resection like quadrantectomy, followed by whole breast irradiation [34–36]. However, for more advanced disease, and a number of other indications, such as inflammatory breast cancer, intraepithelial neoplasia not amenable to breast-conserving surgery, or local recurrence after breast conserving surgery, mastectomy is necessary. At the same time, the mastectomy operation continues to undergo modification as part of the shift to less extensive but oncologically adequate procedures that are more technically demanding than the traditional mastectomy procedures. 

### 5.1. Skin-Sparing Mastectomy

Mastectomy with skin preservation was first applied by Freeman to two patients with benign breast disease [37]. Toth and Lappert [38] were the first to report a skin-preserving mastectomy for breast cancer that required considerable preoperative planning of the incisions in order to maximize skin preservation, and thereby facilitate breast reconstruction—whose aesthetic outcome depends considerably on the quantity of breast skin remaining. Their operation consisted of breast gland removal, removal of the nipple-areola complex, biopsy scar, and skin overlying the cancer (if superficial) with preservation of remaining skin and inframammary fold. 

The benefits of skin-sparing mastectomy are that it reduces postmastectomy deformity, allows better breast shape after reconstruction, minimizes residual scarring, and reduces the area of skin necessary on myocutaneous flaps. It also reduces the need for contralateral breast surgery to achieve symmetry [39]. 

Doubts remain about oncological safety of skin-sparing mastectomy, particularly since randomized controlled studies have not been conducted, and residual breast tissue often remains in the skin envelope [40–42]. However, even in cases undergoing conventional mastectomy, residual breast tissue is present on the skin flaps (excluding nipple-areola complex) in around 23% of cases [43]. Furthermore, retrospective studies with followup ranging from 35 to 70 months suggest no differences in recurrence or survival rates between patients undergoing skin-sparing mastectomy with reconstruction and those undergoing conventional mastectomy [44–51]. 

The most common complications of skin-sparing mastectomy are skin necrosis, infection, and hematoma [52–58]. Infection and hematoma occurs in 2%–19% of cases [52–57]. Skin flap necrosis occurs in about 11% of cases when there are no risk factors [52]. Similar rates of necrosis are observed after modified radical mastectomy [52]. Smoking is a major risk factor for necrosis [52]. Other factors that increase the risk of necrosis are previous breast irradiation, diabetes, and high body mass index [52]. These complications do not usually delay the administration of adjuvant therapies [59–61]. Reduced skin sensitivity occurs in around 65% of patients [62].

Indications for skin-sparing mastectomy are BRCA1/2 mutation, intraepithelial neoplasia (DIN or LIN), particularly when the lesion is extensive, multicentric or recurrent, and early-stage breast cancer for which breast-conserving therapy is not suitable [63]. Some of studies support the use of skin-sparing mastectomy following neoadjuvant treatment, for recurrence after breast-conserving surgery, and malignancy after additive mammoplasty [64–66].

Skin involvement by the tumour is an absolute contraindication for skin-sparing mastectomy. Relative contraindications are adjuvant radiotherapy, smoking, previous irradiation, high body mass index, and delayed reconstruction [52, 67–71].

### 5.2. Nipple-Sparing Mastectomy

Nipple-sparing mastectomy is a refinement of skin-sparing mastectomy in which the nipple-areolar complex is spared. Once again, its aim is to improve the aesthetic outcome of the reconstruction, which is usually performed immediately [72]. Nipple sparing mastectomy can be associated with intraoperative radiotherapy to the nipple-areolar complex [72, 73]. One intraoperative irradiation technique (ELIOT) employs electrons at a dose of 16 Gy directly to the nipple-areolar complex. A combined aluminium and lead disc is placed under the complex and over the chest to protect the chest wall [73].

Contraindications for nipple-sparing mastectomy are central quadrant tumours, clinical evidence of nipple-areolar complex involvement, bloody nipple discharge, palpable tumours less than 1 cm from the nipple, and micro-calcifications or other radiological alterations near the nipple [74]. Frozen retroareolar tissue is examined intraoperatively and if positive for malignancy nipple sparing is not performed [72–74].

Petit et al. [73] reported on 289 patients who underwent nipple-sparing mastectomy with intraoperative radiotherapy (ELIOT) to the nipple-areolar complex. Three percent of their patients had total necrosis of the nipple-areolar complex, an additional 9.5% had partial necrosis, and the entire complex had to be removed in 4.6%. After 1 year of followup, cosmetic outcomes were rated good by 82.3% of patients and by 84.8% of surgeons. Radio dystrophy occurred in 7.5%. Nipple-areolar complex sensitivity returned partially in 48%. No local recurrence occurred beneath the preserved nipple-areolar complex although followup was short [73].

As with skin-sparing mastectomy, there is concern that retaining the nipple-areolar complex increases the likelihood of disease recurrence; however, the available experience suggests that this is not the case [72–76]. To achieve optimal cosmetic results with oncologic safety, nipple-sparing mastectomy should be performed on only carefully selected patients [74, 75].

### 5.3. Prophylactic Risk-Reduction Mastectomy

Five-to-ten percent of breast cancers occur in women with a deleterious mutation in BRCA1, BRCA2, or other breast cancer-associated genes [77, 78]. BRCA1 and BRCA2 mutations are quite penetrating: the cumulative risk of breast cancer is about 65% in BRCA1-mutation carriers (by age 70) and about 45% in BRCA2-mutation carriers [79, 80]. Mastectomy has long been proposed as a means of avoiding breast cancer in women with BRCA1 and BRCA2 mutations. Meijers-Heilboer et al. studied the oncological results of risk-reduction mastectomy in 139 persons with these mutations, randomizing them to breast cancer screening or reducing risk mastectomy [81]. After an average followup of 3.0 years, breast cancers occurred in 8 screening group women but in none of the mastectomy group [81]. However, mastectomy does not completely eliminate the risk of breast cancer [82–84]. Deleterious mutation carriers considering prophylactic mastectomy should be evaluated by a multidisciplinary team consisting of geneticist, oncologist, breast surgeon, plastic surgeon, and psychologist [85, 86].

## 6. Reconstructive Surgery after Mastectomy

Options for breast reconstruction after mastectomy consist of autologous transplants, implants, and combinations of both. Autologous breast reconstruction employs both pedicle-based and free flaps. Local pedicle flaps are latissimus dorsi flaps and transverse rectus abdominis myocutaneous (TRAM) flaps. Free flaps include the deep inferior epigastric perforator (DIEP), free TRAM flaps, and gluteal artery perforator (GAP) flaps.

The latissimus dorsi flap was introduced by Schneiders in 1977 and produces satisfactory aesthetic results [87]. Endoscopy can be used to mobilize latissimus dorsi flaps and minimize dorsal scarring. This flap can also be used in association with implants to improve cosmetic results [39].

The DIEP flap, originally proposed by Holmstrom in 1979, makes large-volume transfer possible, with preservation of the abdominal muscles and aponeurotic layers, [88] and reduces complications such as muscular weakness, bulging and hernia; however, the operating time is longer than for other breast reconstructions.

The TRAM flap was introduced by Hartrampf et al. in 1982 [89]. Patients report high levels of aesthetic satisfaction with this technique [90]. However, donor site complications occur more frequently than with DIEP flap. The free superior gluteal artery perforator (S-GAP) flap was first used for breast reconstruction in 1995 [91]. It allows transfer of skin and fat without sacrificing the gluteus maximus.

Several types of expanders and prostheses are used for breast reconstruction. The introduction of anatomically profiled implants improved this type of reconstruction so that good breast volume, shape, and symmetry are routinely achieved with low complication rates [39, 92, 93]. 

The choice of breast reconstruction technique depends on the anatomical characteristics of the patient and the skill and experience of the plastic surgeon. The risk of reconstruction failure is higher in smokers, obese patients and, those with large breast [39, 52, 71].

## 7. Conclusions

Mastectomy has evolved from the frankly mutilating but often effective Halsted radical mastectomy to the technically more demanding skin-sparing and nipple-sparing approaches that are increasingly used today. These new mastectomies provide results that are oncologically equivalent to the older operations but make it easier to perform immediate breast reconstructions that achieve good aesthetic results and reduce psychological morbidity. At the same time, mastectomy has been supplanted by surgical approaches that conserve the breast for a large fraction of breast cancer patients in Western countries, in part because breast cancer is diagnosed at a much earlier stage. There is no sign yet that surgery for breast cancer will be supplanted by noninvasive treatments, so the way forward seems to be the development of increasingly sophisticated and demanding surgical procedures that are ever more precisely tailored to the individual patient and typically require the skills of a plastic surgeon as well as the breast surgeon and multidisciplinary team. It seems important to develop ways of reducing the failure and complication rates for the breast reconstruction procedures facilitated by the new mastectomies.
